# Editorial Note

**Published:** 1896-03

**Authors:** 


// The Bristol
### Medico-Chirurgical Journal.

MARCH, 1896.

### EDITORIAL NOTE.

In entering on the work of the fourteenth volume of the *Bristol Medico-Chirurgical Journal*, the Editor is gratified to make the announcement that the Committee of the Bristol Medico-Chirurgical Society has authorised an enlargement of the *Journal* to the extent of sixteen pages in each quarterly number. It has not been thought desirable to make any alteration either in the form or the price of the *Journal*, but the Editor hopes that the addition of sixty-four pages in the year will enable him to make the *Journal* more truly a quarterly representative of the medical life and work of the West of England and South Wales than it has hitherto been.

To this end he has arranged with various representative subscribers to co-operate with the Bristol Committee by acting as local representatives in the principal towns of the district, and he has been able to secure the services of the following subscribers, each of whom undertakes to further the interests of the *Journal* in his own neighbourhood:—

| | |
|---|---|
| Bath . . . . | Dr. Gilbert A. Bannatyne. |
| Cardiff . . . | Dr. Donald Rose Paterson. |
| Exeter . . . . | Dr. Arthur G. Blomfield. |
| Gloucester . . . | Dr. Rayner W. Batten. |
| Newport, Mon. . . | Mr. O. E. Bulwer Marsh. |
| Plymouth . . . | Mr. Walter L. Woollcombe. |
| Swansea . . . | Dr. E. Le Cronier Lancaster. |
| Torquay . . . | Mr. William Odell. |

